# First Case of Brucella Pneumonia in a Lung Transplant Patient: Case Report and Review of the Literature

**DOI:** 10.7759/cureus.8733

**Published:** 2020-06-21

**Authors:** Abdulaziz H Abed, Reem S Almaghrabi, Imran Nizami

**Affiliations:** 1 Medicine and Surgery, Alfaisal University College of Medicine, Riyadh, SAU; 2 Infectious Diseases, King Faisal Specialist Hospital and Research Centre, Riyadh, SAU; 3 Organ Transplant, King Faisal Specialist Hospital and Research Centre, Riyadh, SAU

**Keywords:** lung transplantation, solid organ transplant, brucella, brucellosis, pneumonia, pulmonary infiltrate, serology

## Abstract

Brucella is one of the most common zoonotic diseases worldwide. It is endemic in the Mediterranean basin. Brucella pneumonia is a rare complication of brucellosis that can present with a variety of clinical and radiological manifestations. It was described only once previously in the setting of solid organ transplant.

A 32-year-old female from Saudi Arabia with cystic fibrosis and bronchiectasis presented five weeks after a bilateral lung transplant with fever and cough. Investigation showed high inflammatory markers in addition to a pulmonary infiltrate in the chest imaging. All microbiological workups were negative including bronchoalveolar lavage cultures. Brucella serology was positive and she was started on anti-Brucella therapy which resulted in complete resolution of her symptoms and radiological changes. This case demonstrates an unusual presentation of Brucellosis. It highlights the importance of epidemiology in evaluating post-transplant infections. We reviewed and summarized the literature on brucellosis post solid organ transplant and the various treatment regimens for Brucella pneumonia.

This is the first case report of Brucella pneumonia in a lung transplant patient. Brucella is a rare complication post solid organ transplant but it has a good prognosis.

## Introduction

Brucellosis is one of the most widespread zoonotic diseases in the world and is caused by infection with Brucella species, which are intracellular gram-negative coccobacilli [1]. Brucellosis is an endemic disease in several countries, such as those in the Arabian Peninsula. Saudi Arabia has an infection rate of about 70 per 100,000 people [2]. It is a multi-system disease and symptoms include fatigue, malaise, anorexia, and body aches. Fever is the most common sign [3].

Respiratory system involvement in brucellosis is rare, and the non-specific findings make the diagnosis difficult [4]. Brucellosis in the respiratory system results from inhalation of infected aerosol or through hematogenous spread and it can cause a variety of pulmonary manifestations including pleural effusions, pneumonia, lymphadenopathy, and pulmonary nodules, and it can be found in up to 16% of complicated cases [1]. Brucella infection has been reported in organ transplant recipients and is acquired either as donor-derived infection, blood transfusion-related, or due to a new infection post-transplantation [4]. Here, we report the first case of Brucella pneumonia in a lung transplant patient and review the literature on Brucella pneumonia.

## Case presentation

A 32-year-old female patient known to have cystic fibrosis and bronchiectasis with respiratory failure underwent a double lung transplant at the end of November 2017 under methylprednisolone induction. Her pre-transplant workup is summarized in Table 1.

**Table 1:** Pre-transplant infectious diseases workup CMV: Cytomegalovirus; EBV: Epstein-Barr virus; TB: Tuberculosis; HAV: Hepatitis A virus; HBV: Hepatitis B virus; HCV: Hepatitis C virus; D: Donor; R: Recipient; TMP-SMX: Trimethoprim-sulfamethoxazole.

Test	Results
CMV IgG	D+/R+
EBV	D+/R+
QuantiFERON TB	Negative
HAV	Immune
HBV	Immune
HCV antibody	Negative
Microbiology	Fully susceptible Pseudomonas aeruginosa Achromobacter xylosoxidans susceptible to TMP-SMX

The patient had an uneventful course post-transplant and was discharged two weeks later from the hospital on tacrolimus 7 mg twice daily, mycophenolate mofetil 1 g twice daily, and prednisone 20 mg daily for immunosuppressant medication, and trimethoprim-sulfamethoxazole (800 mg/160 mg) tablets three times per week (TMP-SMX), valganciclovir 450 mg daily, isoniazid 300 mg daily, inhaled amphotericin B and itraconazole for antimicrobial prophylaxis, in addition to pancreatic enzymes.

Five weeks after the transplantation, the patient presented to the clinic for a follow-up visit, during which she reported subjective fever, dry cough, and four kilograms of weight loss since her hospital discharge. Her symptoms were associated with central pleuritic chest pain. She reported shortness of breath during the same period that worsened when lying down, and that improved partially when seated. She reported two brief episodes of chills, with no rigors or night sweat. The patient did not experience headache, neck pain, skin rash, photophobia, abdominal pain, change in bowel habit, dysuria, changed urine color, sputum, use of antibiotics, travel, or contact with tuberculosis patients or animals.

On physical examination, the patient was conscious, alert, and oriented. Her temperature on admission was 37.9°C, heart rate was 89 per minute, blood pressure was 105/62 mmHg, respiratory rate 24/min and oxygen saturation was 96% on a 1-liter nasal cannula. Chest: Not in respiratory distress with vesicular breath sounded bilateral, with decreased breath sounds over the bases with dullness on percussion. Cardiovascular: Normal first and second heart sounds with no added sounds. Abdomen: Soft, lax, non-tender with no organ enlargement, no lower limb edema.

The patient was admitted to the hospital for further examination. Her laboratory investigations on admission are summarized in Table 2.

**Table 2:** Laboratory investigations on second admission ALT: Alanine aminotransferase; AST: Aspartate aminotransferase; CRP: C-reactive protein; ESR: Erythrocyte sedimentation rate; Hb: Hemoglobin; HCT: Hematocrit; INR: International normalized ratio; PT: Prothrombin time; PTT: Partial thromboplastin time; WBC: White blood cells.

	Result	Reference Range
WBC	13.54	3.9-11 x 10^9^/L
Hb	98	11-160 g/L
HCT	0.284	0.32-0.47 L/L
Platelets	422	155-435 x 10^9^/L
PT	14.7	12.3-14.2 seconds
PTT	36.1	30.5-40.4 seconds
INR	1.1	0.9-1.1
Potassium	5.2	3.3-5 mmol/L
Sodium	136	135-147 mmol/L
CRP	153	<3 mg/L
ESR	132	0-15 mm/Hr
Urea	6.1	4.2-7.2 mmol/L
Creatinine	65	64-115 umol/L
ALT	22.9	10-45 U/L
AST	20.1	10-45 U/L
Alkaline phosphatase	80.9	46-122 U/L
Tacrolimus level	13.2	3-15 ug/L

**Figure 1:**
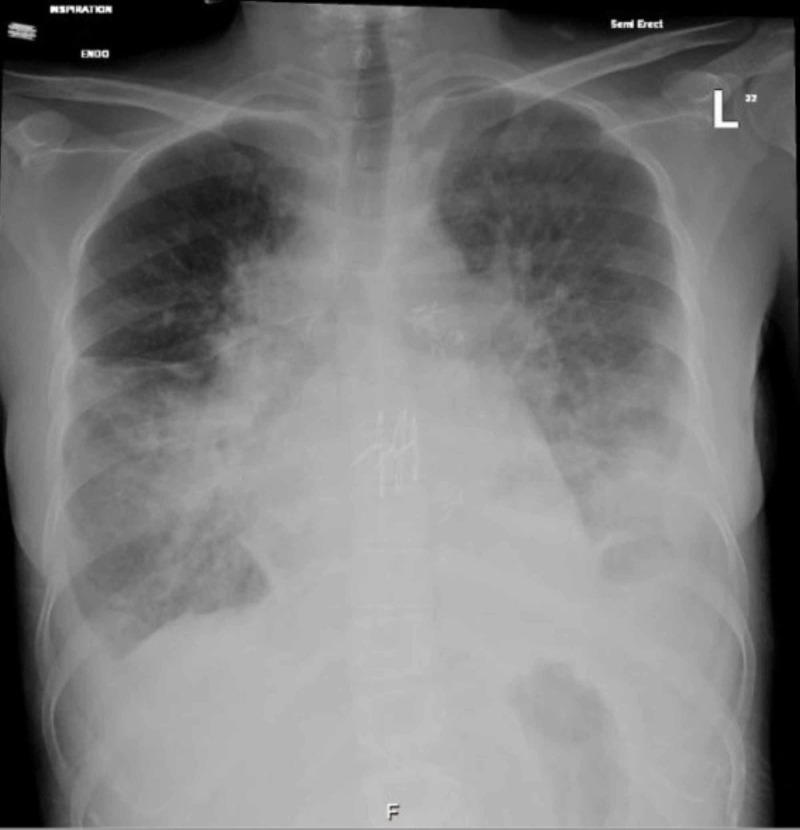
Patient’s initial chest X-ray shows bilateral lower air space and perihilar opacity, bilateral pleural effusion.

**Figure 2:**
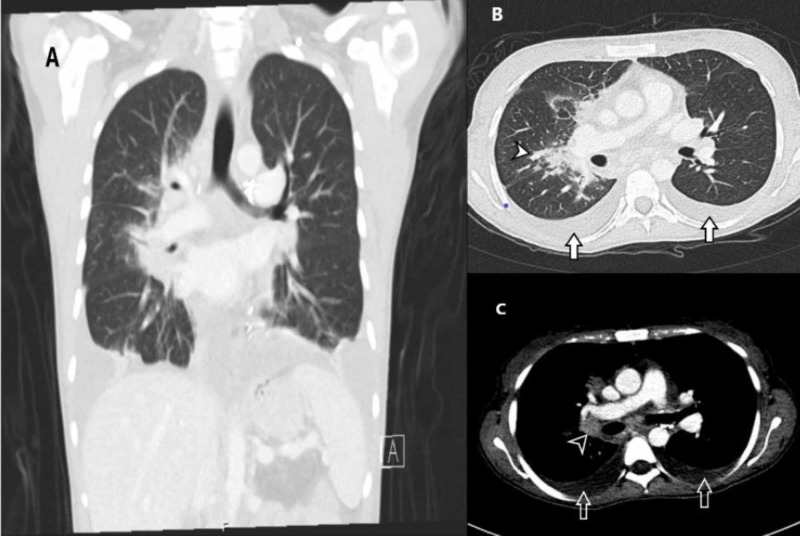
(A) Coronal CT image showing bilateral lower air space and perihilar opacity. (B) Bilateral pleural effusion (Arrows) and right perihilar opacity (Arrowhead). (C) Enlarged enhancing right hilar lymph node (Arrowhead) and bilateral pleural effusion (Arrows).

It showed leukocytosis, mildly elevated platelets, and elevated inflammatory markers. Chest X-ray (Figure 1) and CT scan (Figure 2) of the chest showed bilateral pulmonary infiltrate and peri-hilar opacities. The patient was started on ceftazidime and TMP-SMX based on her prior microbiology results. Blood and sputum cultures were performed and were negative.

**Figure 3:**
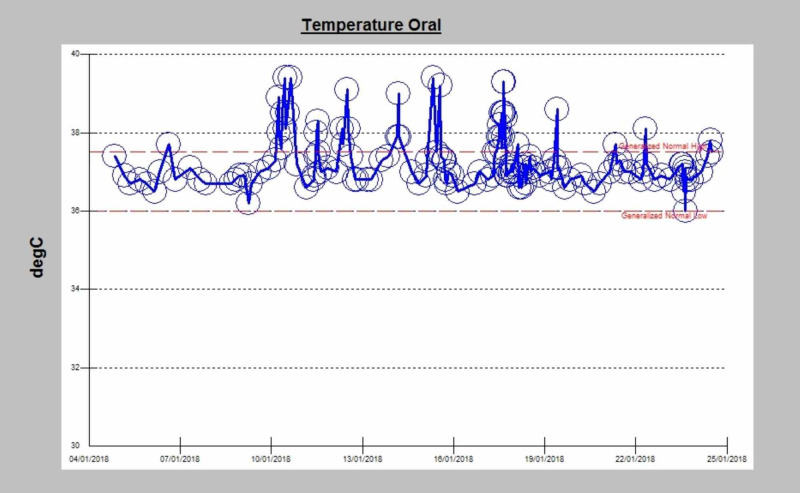
Temperature chart during patient admission and after starting treatment.

Given the presence of a pulmonary infiltrate and mediastinal lymphadenopathy, additional analyses were done that included cytomegalovirus (CMV), Epstein-Barr virus (EBV) viral load, and serum cryptococcal antigen which all came back negative. Three sputum samples for acid-fast bacilli (AFB) stains, mycobacterial cultures, and Mycobacterium tuberculosis polymerase chain reaction (PCR) (GeneXpert MTB/RIF, Cephid, Sunnyvale, California, USA) came back negative. She underwent bronchoscopy for lavage and endobronchial ultrasound biopsies from mediastinal lymphadenopathy twice during admission, and results were negative for malignancy and granulomas, in addition to negative cultures, AFB stains, mycobacterial cultures, and Mycobacterium tuberculosis PCR. The patient continued to have spikes of fever as shown in Figure 3.

**Figure 4:**
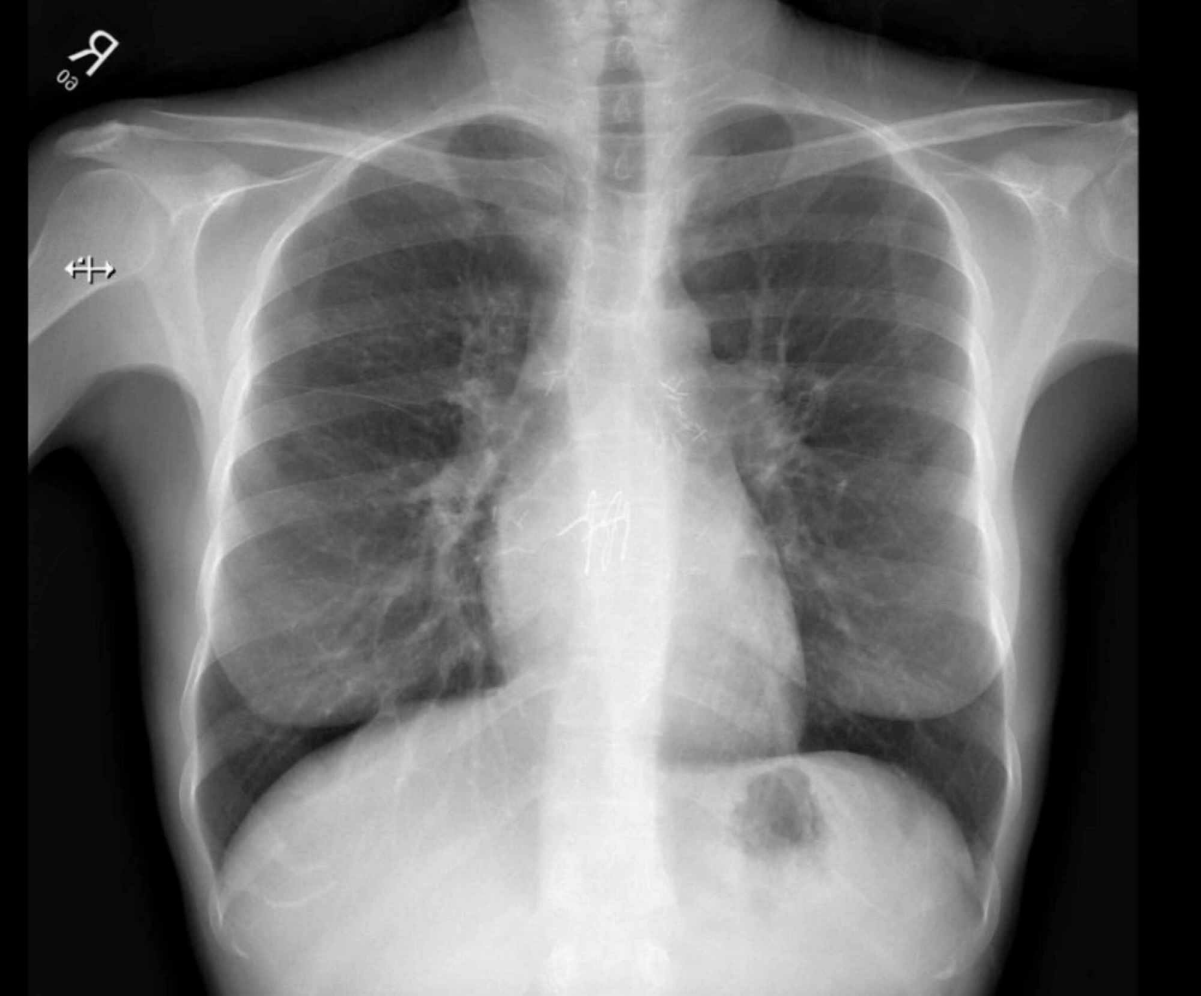
Chest X-ray six weeks after therapy.

As part of the workup for persistent fever, Brucella serology was performed and this came back positive with IgG <1:20 and total antibody 1:1280. The patient was not tested for Brucella prior to transplant, while donor serology and other recipient serology were negative. The patient denied any history of recent animal contact or consumption of raw dairy products but reported remote raw milk ingestion prior to the transplantation which raised the suspicion for an infection that was acquired prior to transplant, and that started to show clinical signs and symptoms after transplantation. The patient was started on streptomycin 1 g daily for two weeks and doxycycline 100 mg twice daily for three months. Her repeat chest X-ray was performed six weeks after start of treatment. The previously observed infiltrates and opacities had disappeared (Figure 4).

## Discussion

This is the first reported case of Brucella pneumonia in a patient post lung transplantation. The patient presented with classical symptoms of Brucella that included high-grade fever and weight loss [5]. A donor-derived infection was ruled out with negative Brucella serology and blood culture from the donor at the time of the organ procurement. Human to human transmission of brucellosis has been reported to occur via blood transfusion, hematopoietic stem cell transplantation, and vertical transmission [6]. There are no reported cases of donor-derived Brucellosis after solid organ transplantation, which may be due to underreporting.

There are a few reported cases of Brucella infection post solid organ transplantation, mostly in renal transplant patients [7-10], liver transplant patients [11-13], and in one cardiac transplant patient [14]. These cases are summarized in Table 3.

**Table 3:** Brucellosis cases in solid organ transplant recipients reported in the literature.

Age in years	Organ transplanted	Time post-transplant	Risk factors	Presentation	Diagnosis of Brucella	Treatment	Duration of therapy	Reference
41	Kidney	3 years	Not reported	Fever and weakness	Blood culture	Doxycycline, TMP-SMX, rifampin	6 weeks	Bishara et al. [15]
56	Kidney	3 years	Remote history of raw dairy product consumption	Fever and confusion	Serology	Doxycycline, rifampin	6 weeks	Yousif and Nelson [7]
58	Kidney	3 years	Raw cheese consumption	Fever and arthritis	Blood/synovial fluid culture	Doxycycline, rifampin, ciprofloxacin	Not reported	Einollahi et al. [8]
15	Liver	2 months	Lives in endemic area	Fever and poor appetite	Serology	Doxycycline, rifampin	8 weeks	Polat et al. [13]
58	Kidney	3 years	Traveled to endemic country	Fever, chills, and sweating	Blood culture	Tigecycline IV, Minocycline, TMP-SMX	2 weeks 3 months	Ting et al. [9]
39	Liver	2 years	Not reported	Fever and poor appetite	Blood culture and serology	Rifampin, TMP/SMX	8 weeks	Xie et al. [16]
7	Liver	2 years	Lives in endemic area & raw cheese consumption	Fever	Serology	Rifampin, TMP/SMX	3 months	Islek et al. [11]
12	Liver	5 years	Lives in endemic area	Fever and hip pain	Blood culture and serology	Doxycycline, rifampin	8 weeks	Sutcu et al. [12]
20	Kidney	4 months	Occupation	Fever and cough	Serology	Rifampin, doxycycline	6 weeks	Ay et al. [17]
63	Kidney	8 years	Lives in endemic area	Fever	Blood culture and serology	Ciprofloxacin, doxycycline	2 weeks 6 weeks	Inayat et al. [10]
51	Heart	3 months	Farmer with animal contact	Fever, chills, and leukopenia	Serology	Doxycycline, TMP-SMX	3 months	Nair et al. [14]

All cases presented with fever and the majority also had high inflammatory markers. Direct animal contact and/or raw dairy product consumption was reported in a few cases but being from highly endemic areas was the most common risk factor reported in the setting of organ transplantation.

Pulmonary involvement in brucellosis is rare. The largest reported case series came from Turkey in 2003 (37 cases) [18], 2005 (11 cases) [4], and 2014 (133 cases) [19]. Other reported case reports were post renal and liver transplantation [20]. Fever and cough were the two most common presenting symptoms. Extra-pulmonary involvement was present in 27%-75% of the patients. The radiological manifestations varied, with lobar infiltrate/consolidations as the most common presenting radiological feature. Other presentations such as pulmonary nodules or pleural effusion have also been reported. The treatment regimen was not consistent across the reported cases of Brucella pneumonia. A systematic review of the treatment of Brucella pneumonia found that a combination of doxycycline and rifampin is the most commonly used regimen followed by doxycycline and aminoglycosides. All treatment regimens resulted in an excellent prognosis with mortality reported to be <1% [19].

## Conclusions

In conclusion, Brucella is a rare complication post solid organ transplant. The small number of reported cases could be due to underreporting. Brucella pneumonia is a well-known manifestation of Brucellosis. In highly endemic areas, Brucella pneumonia should be considered as a differential diagnosis of pneumonia, especially in post solid organ transplant patients. A combination of the commonly used doxycycline and rifampin or doxycycline and aminoglycosides showed an excellent prognosis with a very low mortality rate.
